# Supplementary material for: LandS: Vegetation modeling based on Ellenberg's ecological indicator values
Source: MethodsX. 2023 Nov 13;11:102486. doi: 10.1016/j.mex.2023.102486 (PMC10709510; doi:10.1016/j.mex.2023.102486)
Supplement: Supplementary file 1 [file mmc1.docx]

LandS -Model – ODD

ODD Version 1

**Content**

[1 Purpose and patterns 5](#_Toc142491385)

[2 Entities, state variables and scales 6](#_Toc142491386)

[3 Process overview and scheduling 10](#_Toc142491387)

[4 Design concepts 12](#_Toc142491388)

[4.1 Basic principles 12](#_Toc142491389)

[4.2 Emergence 14](#_Toc142491390)

[4.3 Adaptation 14](#_Toc142491391)

[4.4 Objectives 15](#_Toc142491392)

[4.5 Sensing 15](#_Toc142491393)

[4.6 Interaction 16](#_Toc142491394)

[4.7 Stochasticity 16](#_Toc142491395)

[4.8 Observation 17](#_Toc142491396)

[5 Initialization 17](#_Toc142491397)

[5.1 Initialization after pre-configuration 18](#_Toc142491398)

[5.2 Pre-configuration 20](#_Toc142491399)

[5.2.1 Necessary data obtained from a pre-configuration 20](#_Toc142491400)

[5.2.2 General approach for the pre-configuration and calibration of the model 21](#_Toc142491401)

[6 Input data 31](#_Toc142491402)

[7 Submodels 31](#_Toc142491403)

[7.1 Herbaceous vegetation 31](#_Toc142491404)

[7.1.1 Purpose 31](#_Toc142491405)

[7.1.2 Process overview and scheduling 32](#_Toc142491406)

[7.1.3 Herbaceous vegetation submodels 33](#_Toc142491407)

[7.2 Woody vegetation 36](#_Toc142491408)

[7.3 Disturbance by wildlife 37](#_Toc142491409)

[8 References 37](#_Toc142491410)

**List of figures:**

[Figure 1. General model concept. 9](#_Toc142491380)

[Figure 2. Processes and scheduling. Process names correspond to those of the submodels described in detail in Section 7. 10](#_Toc142491381)

[Figure 3. An example decision tree for deriving vegetation types from species composition, the name of species is referring to their simulated cover, first published by Siehoff et al. (2011). 23](#_Toc142491382)

[Figure 4. Screenshots showing parts of the output GUI of two different simulation runs: at the start of the calibration process (left) and after calibration (right) with the dynamic equilibrium of the examined community. 30](#_Toc142491383)

[Figure 5. Control functions for site conditions (A) and land use (B) at different species EIVs. (A) Control functions are calculated here for regular species EIVs with a slope of 1, and for indifferent species EIVs with a reduced slope of 0.3 and a reduced optimum of 0.6. (B) There are no indifferent species for the land use. 35](#_Toc142491384)

**Tables**

[Table 1. Model components. Simulation settings, state variables, and parameters of the model. 6](#_Toc142491370)

[Table 2. Overview of the elements for the initialization. 18](#_Toc142491371)

[Table 3. Global parameters specified in the GUI for the initialization. 19](#_Toc142491372)

[Table 4. Landscapes with existing preliminary model setups 21](#_Toc142491373)

[Table 5. Different types of initialization tables depend on the vegetation type. 21](#_Toc142491374)

[Table 6. Land use scenarios with their corresponding utilization intensity were first published by Siehoff et al. (2011). 24](#_Toc142491375)

[Table 7: Advantages and disadvantages of the possible methods for the derivation of Ellenberg site EIVs. 25](#_Toc142491376)

[Table 8. Overview of how soil map information is used to derive site EIVs. The parameters provided refer to the information available in the German soil map of NRW, BK50 (GD NRW, 2023; Schrey, 2014). 28](#_Toc142491377)

[Table 9. Categorization of observable patterns to derive exemplary start values for calibration. 31](#_Toc142491378)

[Table 10. Equations used in the herbaceous vegetation submodel 33](#_Toc142491379)

This model description follows the ODD (Overview, Design concepts, and Details) protocol (Grimm et al. 2020). Earlier versions of the model were published separately, focusing on different aspects of the landscape: herbaceous vegetation and early woody encroachment. While herbaceous vegetation is a mandatory element of the model, wood encroachment is optional. As the model extension in this publication has been developed for herbaceous vegetation only, the description for woody encroachment part only provides an overview. For more information on woody vegetation, see Hudjetz et al. (2014).

Following Supplement S3 of Grimm et al. (2020), we provide a “nested ODD”, where the submodel representing herbaceous vegetation is described by an ODD description of its own. In addition, we follow Supplement S4 of Grimm et al. (2020) on “ODD of modified models” by using different font colors to distinguish elements of the model that are new or from the two earlier version. The color code used is:

- M1: ODD with a focus on grassland (*herbaceous vegetation)*, GraS-Model (Siehoff, 2011; Siehoff et al., 2011).
- M2: ODD with a focus on early wood encroachment (*woody vegetation)*, WoodS-Model (Hudjetz et al., 2014; Siehoff, 2011).
- M3: Current version. Nested ODD, with a focus on the model extension for the herbaceous vegetation.

The original Delphi code implementing the model has been overhauled. It is now strictly object-orientated. All objects and processes described in the text correspond to objects and methods in the source code. The executable version of the model is available online at <https://github.com/gaiac-eco/LandS>.

# Purpose and patterns

*Purpose.* The main purpose of this model is to illustrate and predict vegetation succession in semi-natural landscapes for small-scale applications, such as a national park. Therefore, the model simulates the dynamics of the vegetation in a landscape mosaic and includes competition among herbaceous species as well as early wood encroachment. To make the model applicable to different landscapes, the modeled plant species from the data input files require calibration in a pre-configuration process. The model can be used to develop different management scenarios for situations where the landscape has changed or where changes are imminent. Examples include intended/planned changes in land management in protected areas, or action plans following severe storms that have created windthrow areas in forests. Ultimately, the model is intended as a decision support system (DSS) for stakeholders involved in the management of these landscapes.

*Patterns*. In evaluating the model, we distinguish between two types of vegetation patterns: equilibrium vegetation communities and intermediate state vegetation communities. Equilibrium patterns are observable in reality and can be derived from field data, expert knowledge, or the literature. Intermediate vegetation communities occur after a change in environmental conditions, such as abandonment. These communities transition between equilibria, are usually short-lived, and are not necessarily realistically simulated. We thus focus on the final outcome of succession, rather than on all the details of the transient dynamics, which are known to be often idiosyncratic anyway. If time series of vegetation data are available, the transition stage can also be evaluated.

# Entities, state variables and scales

The model contains the following entities: the *landscape* representing the global environment, grid *cells,* and two types of plant entities for the *herbaceous* and *woody vegetation*. The entities and their state variables are listed in Table 1.

Table 1. Model components. Simulation settings, state variables, and parameters of the model.

| Model component | Symbol | Default / Initialization value | Unit |
| --- | --- | --- | --- |
| Landscape (global environment) |  |  |  |
| Number of cells |  | 10 x 10 | – |
| Cell size | A | 100 | m^2^ |
| Number simulation years | y | 10 | a |
| Time steps per year | t | 365 / 52 | d / w |
| Cell |  |  |  |
| X-Y-coordinates |  | 0,0 | – |
| Type of cell, to be modeled or not to be modeled |  | Yes/No | – |
| Site Ecological Indicator Value for light, temperature, moisture, reaction, nutrients | site EIV_L/T/M/R/N_ | ∈ [1, …, 9] ^1^ | [–] |
| Utilization intensity for cutting, grazing, trampling | I_C/G/TR_ | Scenario specific^1^; ∈ [0, …, 100] | – |
| Available space | AS | – | m² |
| Vegetation type | VT | Study site/landscape specific; depending on observations | – |
| Herbaceous vegetation with grass and forb species (Study site/landscape specific) | | |  |
| Species ecological indicator value for cutting, grazing, trampling | species EIV_C/G/TR_ | ∈ [1, …, 9] ^2^ | – |
| Species ecological indicator value for light, temperature, moisture, reaction, nutrients | species EIV_L/T/M/R/N_ | ∈ [1, …, 9] ^2^ | – |
| Maximum growth rate | g_max_ | ∈ [0, …, 10] ^1^ | a^-1^ |
| Factor for self-regulation | F_S_ | ∈ [0, …, 15000]^1^ | – |
| Cover | c | For initialization depending on observations; later simulated | m^2^ |
| Woody vegetation with bush and tree species (Study site/landscape specific), optional and not used in the current model setup. | | | |

^1^See the corresponding initialization section for further instructions on selection or calibration. ^2^According to Ellenberg et al. (1992), Dierschke (1994), and Briemle et al. (2002) within a maximal change of +/- 0.2

The ***landscape*** component contains the spatiotemporal information of the model, and deals with the simulation, i.e. iterations over years and time steps. The usual cell size is 10m × 10m, but it can be adjusted if necessary. The total size of the simulated area can be large, but it depends on the given study site. The maximal size simulated so far was about 1,500 ha, i.e. 150,000 cells of 100m² over a simulation period of 100 years, calculated in daily time steps. The landscape uses closed boundaries that represent the spatially explicit landscape derived from observations and mapping. In the landscape grid, modeled cells are typically embedded in an environment of unmodeled cells, which are assumed to be stable landscape elements that are not affected by succession. The general idea is that in order to produce recognizable maps and scenarios, it is helpful to include surrounding structures, such as roads or forest areas, that shape the landscape but are either not involved in the short-term succession or are only indirectly involved (e.g., forest areas can still serve as a seed source). These surrounding structures also act as a buffer around the modeled area, so the model is not dependent on periodic boundary conditions.

Each ***cell*** is embedded in the landscape grid and characterized by its *X/Y coordinates*. Cells are divided into two *types*: cells to be modeled and cells not to be modeled. A modeled cell is further characterized by its environmental factors (i.e., light, temperature, soil moisture, reaction (soil pH), soil nutrients, and land use intensity of cutting, grazing, and trampling), which are determined by the study site and potential scenarios, see Section 5.2.2.2 ‘Site parameterization – site EIVs’ for a description on how to obtain values for these factors. These environmental factors are integrated either as *site-specific Ecological Indicator Values* (*site EIVs*) or as *Utilization Intensity* (I), see Section 4.1 Basic Principles for an in-depth explanation of the concept of EIVs. In each cell, the growth of the vegetation entities is modeled (Figure 1). The whole community within a cell cannot occupy more than 100% of the area. Therefore, the *available area*, i.e. the area not yet covered by vegetation, in a cell is defined as a state variable. Each cell is further characterized by the state variable *vegetation type*, which represents the plant community in a cell. The vegetation type is used to initialize the vegetation entities. It is also a summarizing endpoint for convenient visualization and comparison with field surveys.

The model contains two types of plant entities, *herbaceous species* (i.e., grass and forb species) and *woody species* (i.e., tree and bush species). In the model, we use a hybrid approach for them: an individual-based model (IBM) for woody species and a compartment model with difference equations for the herbaceous species. While woody species are further divided into two types according to their life form (bushes and trees), grass and forb species are modeled equally at the population level in a cell. Therefore, each cell can contain only one compartment of each herbaceous species, but more than one woody individual, depending on the cell size and the size/age of the individuals.

***Herbaceous vegetation****.* Herbaceous plants are modeled as compartments in a cell, with the state variable *cover* representing the abundance of each species. In general, each herbaceous entity is characterized by species-specific, static state variables for its preference or tolerance to various environmental factors, i.e. species-specific Ecological Indicator Values (*species EIVs*). These values are derived from the phytosociological literature; see Section 5.2.2.3 ‘species parameterization’ on how and where to obtain these values. Other state variables are calibrated during the preliminary model setup, i.e. a *maximum growth rate* and a *self-regulation factor* (F_S_).

Because of the compartmental model, the herbaceous layer is modeled as a homogeneous entity in each cell. This does not allow for fine-scale heterogeneity, which is important for plant coexistence (Silvertown, 2004). Therefore, F_S_ is used to substitute for microsites that are unfavorable for the dominant species. The factor makes room for more inferior species by limiting the species to a maximum cover and preventing the best-adapted species from taking over all the space. Plants with a low F_S_ value are reduced in growth at a lower cover than species with a high F_S_‑value.

While it is possible to model all existing species in the herbaceous layer, this could lead to overparameterization and hinder model calibration and output. In these cases, a set of representatives can be selected. Representatives are either a single plant species or a plant group consisting of species with similar ecological characteristics. For further details about the choice and parameterization of representatives, see species parameterization (5.2.2.3).

***Woody vegetation.*** Optional and not used in the current model setup.

**Scale**. Time in the model is run in years, with the time steps in a year set to either one-day or one-week. Thus, the user can specify the number of years, e.g., 10 or 100 years, and run a simulation with either 52 or 365 time steps per simulation. In a simulation, most of the processes will occur at every time step (e.g., growth), while some will occur only at certain times of the year, such as certain events in the life cycle of trees. The timing of such annual events is chosen to be close to reality (e.g., seed dispersal in summer). However, species-specific variability is not integrated. Furthermore, vegetation development is not seasonal or growing season dependent, i.e. there is no vegetation dieback in winter.


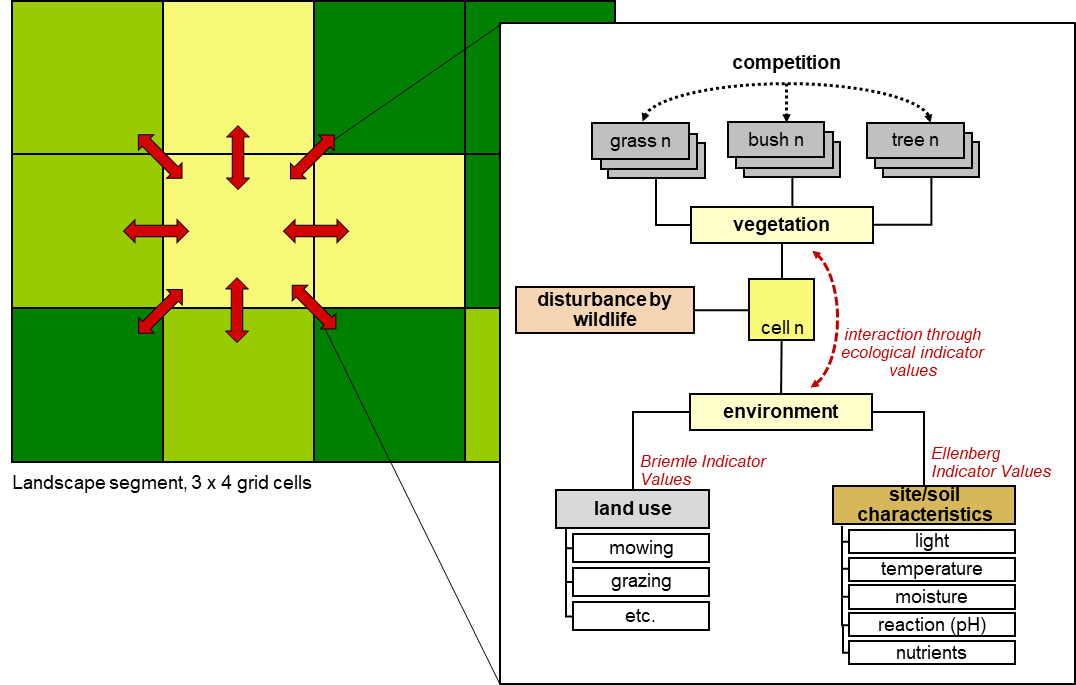


Figure 1. General model concept.

# Process overview and scheduling

Landscape development is modeled by nesting processes at two spatial scales: the year and daily or weekly time steps within the year. All cells are recalculated from the upper left to the lower right at each time step. To account for interactions between neighboring cells, the relevant variables are stored and continuously updated throughout the cell cycle. Processes based on these neighbor interactions, which ultimately update the state variables, are then executed in separate cell cycles. The processes are scheduled as shown in Figure 2.


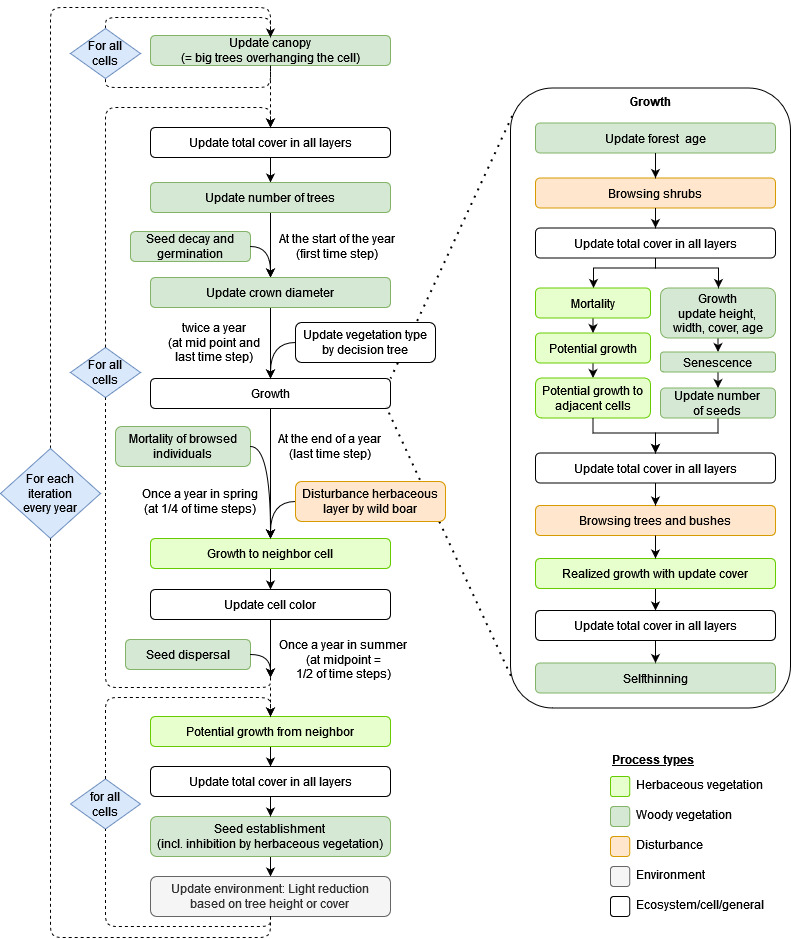


Figure 2. Processes and scheduling. Process names correspond to those of the submodels described in detail in Section 7.

*Herbaceous vegetation*. For the forbs and grasses*,* we considered processes of vegetative growth, mortality and interspecies competition within a cell, and vegetative growth from adjacent, neighboring cells. In each time step, the following processes for the herbaceous layer are executed in the given order. They are divided into two cell cycles. The process names correspond to those of the submodels that implement them, which are described in detail in Section 7.

First cell cycle:

- Update visualization, i.e. vegetation type by a decision tree using the cover of all entities (not in every time step, only twice a year)
- Update of the cover of all herbaceous compartments (entities) in a cell by the dynamic growth and competition model, consisting of:
  - Mortality, which updates the cover of all compartments and the available space in the cell by the increased amount.
  - Potential growth in a cell, which updates the potential growth of each entity.
  - Potential growth to adjacent cells, which determines the target cell and the amount of potential ingrowth to the target cell, and updates the potential growth in the current cell by the reduced amount.
  - Realized growth (competition model), which uses the available space and the potential growth of all entities (in a cell and of its neighbors from the previous time step) to update the cover of each entity.
- Update the storage variable of the amount of potential growth to neighboring cells in a temporary array for the next time step.

In a second cell cycle, the sum of potential growth from neighbors in each cell is updated.

*Woody vegetation.* Optional and not used in the current model setup.

# Design concepts

## Basic principles

Grid-based approach

The model consists of two vegetation submodels that are embedded in a raster-based landscape. This raster-based approach allows the initialization with spatially explicit input data of the actual landscape, integrating the initial vegetation composition and the resulting neighborhood interactions, which are crucial for the course of the succession in a given landscape (Ellenberg & Leuschner, 2010; Müller & Rosenthal, 1998; Prach & Řehounková, 2006; Schreiber, 1997; Smit & Olff, 1998). Moreover, the approach allows the simulation of various management regimes on distinct areas of the modeled landscape. Further, we chose a detailed spatial scale for the grid cells to avoid artifacts, which might result from a wider grid (Bithell & Macmillan, 2007).

Ecological Indicator Values

In our approach, the interaction of plant species with their environment is based on the plant sociological concept of Ecological Indicator Values (EIVs) often referred to as Ellenberg indicator values, which is one of the most prominent examples of EIVs (Diekmann, 2003; Zolotova et al., 2023). EIVs are ordinal scales of environmental gradients such as light intensity, soil moisture, or nutrient availability that have been defined based on large, empirical datasets. The values do not represent physiological requirements, but rather the ecological preferences of plant species along these gradients. Thus, these values not only integrate the relationship of plants to environmental factors, but also capture to some extent distributional and competitive relationships. They are referred to here as *'species EIVs'*. The actual growth, and hence cover, of a species then depends on how well the species EIVs match the corresponding environmental factors, the *'site EIVs'*. Spatially explicit changes in site EIVs are used to simulate management or disturbance scenarios.

Coupling of compartment and individual-based modeling for plants

In a landscape, different scales of vegetation need to be considered. In the case of grasses and forbs, clonal growth and size make it difficult to distinguish individuals in the field. Therefore, grasses and forbs are perceived more as the sum of these plants, whereas bushes and trees can be distinguished as single individuals in the landscape. To account for the activity of these plants at these different scales, we choose different modeling approaches for the vegetation layers: an IBM for woody species and a compartment model with difference equation for the grass and forbs. This type of hybrid approach is considered a powerful tool for environmental modeling (Gross & DeAngelis, 2002; Vincenot et al., 2011). For example, the model of native beech forests, BEFORE (Rademacher et al., 2004) uses cover to describe seedlings and young trees up to 3 m, and individuals to describe canopy and sub-canopy trees.

Vegetation types

We use the plant sociological concept of plant communities to categorize vegetation patterns that correspond to different combinations of environmental factors (Dierschke, 1994). In the model, these plant communities are referred to as vegetation types. Species composition and abundance (cover) can be used to describe and distinguish vegetation types. A distinction is usually made between differential and companion species. Differential species are characterized by their relative abundance and consistent occurrence in a given plant community. Companion species, on the other hand, are species that occur in a plant community but do not play a constant or dominant role. This concept allows aggregating the simulated cover of the plant species, the primary model output, into the vegetation type corresponding to that species composition. For this reason, the model is equipped with a decision tree that derives a vegetation type based on the simulated cover for each cell. This model output can then be linked to a GIS to visualize detailed raster maps, which is a precondition for the application by stakeholders who want to apply the model to existing landscapes (Rammig, 2005).

Initialization and Calibration

The initialization and calibration of the herbaceous vegetation are based on two general ecological concepts, the dynamic equilibrium of plant communities (Knapp, 1974) and space-for-time substitutions (Pickett, 1989). Plant communities in equilibrium in the sense of Braun-Blanquet (1964) include both climax communities and permanent communities (=Dauergesellschaften) that have not reached their climax state but are stable in composition. The space-for-time concept is used to determine the long-term effects of varying environmental conditions on vegetation types by using spatially different sites in the given landscape with varying conditions to substitute for the temporal observations of vegetation succession. For the pre-configuration, the equilibrium vegetation patterns under different environmental conditions of the studied landscape are used to calibrate two species-specific state variables (g_max_ and F_S_). The more vegetation types, and therefore combinations of environmental factors, used in the calibration, the better the plant behavior will reflect different real-world scenarios.

## Emergence

The main outputs of the model are vegetation type maps of the landscape that are based on a decision tree that evaluates the key outcomes of the simulation: the herbaceous plant cover, and the number, age and cover of trees and bushes. These key outcomes emerge from the competition within and between the different vegetation layers in a cell, the life cycle processes of trees and bushes, the disturbance by wildlife, and the effects of the environmental conditions.

*Herbaceous vegetation.* The competitive vigor of herbaceous species emerges from their growth rate in relation to that of the other herbaceous species in the community of a cell, and depends directly on the environmental conditions. The cover of species over the simulation time results from their initial cover, their growth under competition, and the ingrowth from adjacent cells (neighborhood interactions). Spatial and temporal vegetation dynamics then emerge from the cover of single species.

*Woody vegetation.* Optional and not used in the current model setup.

## Adaptation

*Herbaceous vegetation.* Species have three behaviors that indirectly represent adaptation to their own state and that of their environment: the potential to grow into neighboring cells, the reduction of their growth under unfavorable environmental conditions, and a growth limit. Growth into neighboring cells is modeled as direct objective-seeking: if an entity’s cover is above a threshold, it has the potential to grow into an adjacent cell (see Objectives and Stochasticity). Growth reduction under unfavorable conditions is modeled as indirect objective-seeking: if a species EIV is not equal to the corresponding site EIV in a cell its growth factor is reduced (see Sensing and Calculation of growth rate 7.1.3.2). The growth limit is also modeled as indirect objective-seeking. The species have two calibrated parameters, g_max_ and F_S_, which modify the growth rate. The values of the parameters are estimated for each species through an iterative calibration process (see 5.2.2.3 species parameterization for a detailed description). The objective measures during calibration in the pre-configuration process are species assemblages with specific species cover that have been obtained at the study site. These vegetation assemblages differ between environmental conditions, e.g. the specific species cover of a meadow differs from the species cover of a pasture. If the simulated cover for a species during the pre-configuration process is not similar to the objective measure, the g_max_ and F_S_ parameters are adjusted.

*Woody vegetation.* Optional and not used in the current model setup.

## Objectives

*Herbaceous vegetation.* The objective measure of the adaptive behavior ‘growth into neighboring cells’ is the cover of the species. If the cover exceeds the threshold of 1%, a stochastic process is applied to decide whether the entity grows into a neighboring cell or not, and if so, which of the eight neighboring cells is chosen.

*Woody vegetation.* Optional and not used in the current model setup.

## Sensing

*Herbaceous vegetation.* Plant entities can sense the site conditions (i.e., light, temperature, moisture, reaction, and nutrients) and the management regimes (i.e., different types of land use) in their cell. They do this by comparing their own species' EIV with the corresponding site EIV of the cell. The comparison is made in separate control functions that result in a factor that modifies the growth of the species. For site conditions, Ellenberg’s species EIVs indicate the realized niche for a plant species. The further the species deviates from the optimal values, the more its growth is reduced. Species with a lower Briemle species EIVs are more sensitive to a certain form of land use and are more heavily restricted in their growth.

*Woody vegetation.* Optional and not used in the current model setup.

## Interaction

The model includes interactions within and between the different vegetation layers. All plants compete directly or indirectly for space. Herbaceous species compete directly for space within their layer, while woody species, once germinated, grow and use the space indirectly in the herbaceous layer. Large trees can also interact directly with the environment by changing the light conditions.

*Herbaceous vegetation.* Due to interspecific competition, species cannot always realize their full potential growth but have to share the available space with other species. The share of the available area that each species gets is calculated according to the species’ potential growth, which is calculated dynamically based on the species’ current growth rate. The species with the highest potential growth will achieve the biggest share of the available area and will outcompete the other species over time. This approach is similar to the concept of “lottery competition” (Chesson & Warner, 1981).

When trees and bushes are available, certain species in the herbaceous layer can inhibit species-specifically the growth of woody seeds by reducing the number of woody seeds available in a cell.

*Woody vegetation.* Optional and not used in the current model setup.

## Stochasticity

To take natural variation into account, several processes are calculated stochastically:

*Herbaceous vegetation.* Since the raster-based approach can only distinguish between the different covers of species in different cells, and no information about distribution within a cell is given, the spatial dispersal of plants is modeled stochastically. At each time step, it is randomly chosen whether a plant grows into an adjacent cell. The probability (p) of spread to neighboring cells is implemented as a linear function of species’ cover (c), so that the higher the cover, the greater the probability of dispersal to other cells (p = c / cell size). It is randomly chosen, into which one of the adjacent cells the species spreads, as well as the percentage of growth that is transferred to the adjacent cell.

*Woody vegetation.* Optional and not used in the current model setup.

*Disturbance by wildlife*. Used for *woody vegetation*, therefore not relevant for the current model setup.

## Observation

The primary output of a simulation is the cover of each species in each cell. For visualization, the cover of the dominant plants and differential species is used in a decision tree to derive the vegetation type corresponding to the species composition of a cell. These vegetation types can then be used to illustrate the simulation results in detailed raster-maps. The representation as a raster map with the cells as vegetation types resembles the results of a biotope mapping as it is done in reality in nature conservation and thus enables the comparison and transfer of the simulation results. The user can either view the output in the graphical user interface (GUI) or export it to text or CSV files for further analysis or upload to a GIS.

The GUI provides the user with an interactive copy of the simulated landscape, allowing navigation through the simulated years and individual cells. The vegetation type in each cell is indicated by a different color. Different observation tabs provide the user with different information, either based on a selected cell or for the entire simulated area (landscape level). For each cell, the user can toggle between a graph showing the development of each species cover over the simulated time or a pie chart view showing the species cover for a single simulation year. In addition, the user can view cell-defining environmental properties, such as the site EIVs, as well as simple summaries, such as the number of trees and bushes in a cell. For the entire simulated area, the user can also view graphs over time and pie charts, similar to the cell evaluation, but now for the species cover of all cells. These views are particularly useful for calibration and when simulating a homogeneous area. At the landscape level, the user is also provided with a pie chart showing the percentage of vegetation types present.

# Initialization

Each landscape contains specific vegetation communities. While the basic concept of the model is generic and applicable to many landscapes, the model requires landscape-specific (= case-specific) initialization data that define the entities of a simulated landscape, i.e. the species of the herbaceous vegetation and the associated vegetation types. This means that the model must be pre-configured and calibrated for a specific landscape before simulations can be run. While the initialization uses data input files, which can be changed, the core concept of the model depends on using the same species list for a given study area with all its sites. Species competition and species adaptation to different environmental conditions will handle the site-specific variations within the study area. This means, for example, that in previous applications in the Eifel National Park in Germany (Siehoff et al., 2011), meadows, pastures, and abandoned grasslands were all modeled with the same initialization data for species.

## Initialization after pre-configuration

For initialization the model uses imported settings from initialization files, to allow case-specific initialization of different landscapes, and global parameters specified by the user in the GUI. These initialization files can be divided into two types of data: Tables with global settings that define the available plant species and their state variables, and raster-maps with cell-specific settings that define the state variables of the cells (Table 2). The tables with global initialization values are the result of the pre-configuration (see 5.2.2.1 and 5.2.2.3) and are generic for simulations within a landscape, while the cell-specific raster-maps are case-specific and partially exchangeable depending on the simulated scenario. For small test simulations, the cell-specific initialization files can be replaced with user input in the GUI to create a homogeneous cell grid.

Table 2. Overview of the elements for the initialization.

| Element | Source | Type |
| --- | --- | --- |
| Vegetation types and  initial plant cover | Field data, literature on plant communities and vegetation classification (see Section 5.2.2.1). | Global tables |
| Herbaceous species | Field data, expert knowledge, and species EIVs as suggested by Ellenberg et al. (1992), Dierschke (1994), and Briemle et al. (2002); guidance for calibration (see Section 5.2.2.3). | Global table |
| Woody species | Optional and not used in the current model setup. | Global table |
| Vegetation type map | Field data, status quo of the landscape, with cell-specific attribution of number of woody species, when applicable. | Cell-specific  grid map |
| Site EIVs map | Observations, soil map, digital terrain models (DTM), and expert knowledge; guidance for site EIVs (see Section 5.2.2.2). | Cell-specific  grid map |
| Land use map (present and historic) | Observations, aerial photographs, historical maps and interviews with farmers and land users; guidance for site-specific EIVs (see Section 5.2.2.2). | Cell-specific  grid map |
| Decision tree for vegetation types (for output) | Developed based on the available species and possible vegetation types of the current landscape (see Section 5.2.2.1). | Global table |

The GUI allows the user to specify various settings, turn on/off mechanisms, and set the values for global parameters during a simulation. Some of the global parameters (Table 3) modulate the herbaceous vegetation growth model by changing the environmental control functions of the site conditions and weights to balance the effect of all factors. Depending on the landscape studied, each environmental control function of a site condition (light, temperature, soil moisture, reaction, and nutrients) can be adjusted by changing its slope for species with regular EIVs. In addition, the control function can be fitted with a slope and an alternative optimum for species with an indifferent EIV (see submodel 7.1.3.2.1 for more details). Fitting these values is part of the pre-configuration.

Table 3. Global parameters specified in the GUI for the initialization.

| Global parameters in the GUI | Symbol | Default / Initialization value |
| --- | --- | --- |
| Slope for the control functions of each EIV_L/T/M/R/N_ | slope_L/T/M/R/N_ | ∈ [0, …, 2] |
| Slope for the control functions of each EIV_L/T/M/R/N_ for species with indifferent EIVs | slope_red_L/T/M/R/N_ | ∈ [0, …, 1] |
| Optimum for the control functions of each EIV_L/T/M/R/N_ for species with indifferent EIVs | Opt_red_L/T/M/R/N_ | ∈ [0, …, 1] |
| Weights for environmental factors  (light, temperature, moisture, reaction, nutrients, cutting, grazing, trampling) | w_L/T/M/R/N/C/G/TR_ | ∈ [1, …, 20] |

*Landscape and Cells.* The landscape grid is created as a blank slate and then initialized with cell-specific information from raster maps or GUI input fields. Scenarios are later simulated by spatially explicit changes in environmental conditions or management for a cell or area or cells. It also includes global parameters such as calibrated slope and optimum values for the control functions or weights for each environmental factor.

*Herbaceous vegetation.* The entities are initialized with the status quo cover. However, this spatially explicit initialization of herbaceous entities is simplified, because vegetation maps typically consist of mapped vegetation types with a list of plants in each vegetation type. Accordingly, each cell is assigned a vegetation type for which a specific cover per plant species has been defined and provided in the imported global tables.

For simulating secondary succession, such as the development of established plant communities following a change in land use, this approach of starting with the status quo vegetation cover has proven useful. However, to simulate primary succession, i.e. the development of vegetation following disturbance or construction activities that result in larger areas with raw soil conditions, a soil seed bank can be mimicked by initializing all available species in the landscape with a low initial cover, e.g. 0.1%.

Herbaceous entities of the same species only differ in their state variable ‘cover’ at initialization. This depends on the cell and its vegetation type in which they were created. Entities of different species will also differ based on the remaining state variables defined in the imported global tables. EIVs are based on literature values. Species with similar ecological preferences and occurrences may also be similar at initialization.

*Woody vegetation.* Optional and not used in the current model setup.

*Disturbance by wildlife*. Used for *woody vegetation*, therefore not relevant for the current model setup.

## Pre-configuration

### Necessary data obtained from a pre-configuration

The pre-configuration includes the following mandatory elements: vegetation types, herbaceous layer entities, and environmental conditions. The main sources for the pre-configuration are local vegetation relevés from different sites of the study area, covering the different environmental conditions and combinations. They are supplemented by expert knowledge, literature, or historical data (Table 2). The quality and completeness of the vegetation data available from the study area can greatly influence the quality of the pre-configured model, it is important to cover the range of environmental conditions. Detailed instructions for the pre-configuration and the estimation of site-specific EIVs are provided below.

*Herbaceous vegetation*. For vegetation distribution, the cover of each modeled species must be specified for each vegetation type. A cell is then assigned a vegetation type and the corresponding cover for all its entities. The initial vegetation distribution in the simulated landscape can influence the simulation outcome. Species that would be favored by a certain set of environmental conditions, but do not exist in that part of the landscape, might migrate from other parts of the landscape and thus affect vegetation dynamics.

*Environmental conditions*. Site and soil conditions are defined similarly to Ellenberg’s species EIVs on a 9-point scale. Land use is applied with relative values between 0 and 100 for cutting, grazing, and trampling, i.e., all set to 0 for no disturbance.

*Woody vegetation.* Optional and not used in the current model setup.

### General approach for the pre-configuration and calibration of the model

The pre-configuration of the model and the parameter calibration of the chosen entities have a great influence on the model output. Therefore, we describe here the general approach and patterns followed for calibration. These are required for each new landscape to be simulated. Existing model configurations are listed in Table 4.

Table 4. Landscapes with existing preliminary model setups.

| Landscape and site location | Special features | Source |
| --- | --- | --- |
| Mesophilic, extensively used grassland; Eifel National Park, Germany | Based on land use, Briemle EIVs | Siehoff et al. (2011) |
| Windthrow areas in spruce forests; Hochsauerland district, Germany | Based on land use and environmental conditions, Briemle’s EIVs and Ellenberg’s EIVs | Not published |

#### Vegetation types for initialization and evaluation

Vegetation types, a concept from phytosociology (see Section 4.1), are important for the aggregation of information on species composition and abundance at a higher landscape scale. They play two roles in the model. First, they manage the initialization of plant entities, and second, they aggregate the simulated cover of all plants into a single endpoint for visualization. Therefore, we initialize the model with three different global tables: one general, one for the initialization process, and one for the evaluation process (Table 5). Each of these tables must be adapted to the specifics of a new landscape during the pre-configuration.

Table 5. Different types of initialization tables depend on the vegetation type.

| Table | Description |
| --- | --- |
| Vegetation type | Contains all vegetation types of the landscape and whether a type is simulated or not, e.g. roads or field paths are structural elements and are not dynamically simulated. It is required as attribute table for the raster-based vegetation type map. |
| Initialization cover | Contains all simulated vegetation types with the respective initialization cover for each relevant plant entity |
| Decision tree | Contains dominating and districting entities with thresholds to derive the vegetation type from the simulated cover of the plant assemblage |

*Set of vegetation types.* Using field data from a given study area and expert knowledge is the best way to determine the vegetation types of a landscape in the pre-configuration process. First, an analysis of vegetation relevés (or vegetation inventories) and vegetation maps can be used to determine which vegetation types are most prevalent in the study area. In addition, it is important to determine which vegetation types are in a stable state of equilibrium under the current environmental conditions and which vegetation types may be in an active successional process between equilibrium states. These equilibrium states will be the reference for the calibration of species competition parameters (see Section 5.2.2.3). It is also necessary to analyze which environmental factors, such as light availability in forests, soil moisture in arid areas, or soil nutrients in nutrient poor areas, etc., or some combination of thereof, are responsible for the observed patterns in the current landscape. This can then be used to calibrate the weights for site EIVs in the model, and thus the influence, that individual environmental factors have in modifying the growth function of species in a simulation (see Section 7.1.3.2.1).

*Initialization cover.* Simulations typically begin with the vegetation status quo at a study site. However, the real landscape is simplified in that each vegetation type is assumed to have a particular species composition and cover. It is therefore necessary to specify the initial cover of each herbaceous entity for a given vegetation type. This initial cover is also derived from the relevés, e.g. as an average of the mapped plots of a vegetation type. However, it is important to use only plots that represent mostly pure equilibrium states of a vegetation type for the calculation to exclude edge effects due to vegetation plots that are in a transition between states.

*Decision tree.* One of the last steps in the pre-configuration is usually the creation of the decision tree. The decision tree is based on the dominant and differential species that are used in the current simulation and their cover in a cell. The model executes queries using logical conditions such as “IF species 1 > 50% AND/OR species 2 < 5%”. Once a logical condition is satisfied, the corresponding vegetation type is assigned to a cell. It is therefore important to make the conditions specific enough and to allocate them in an ecologically meaningful order, e.g. starting with the dominant species with a high cover, followed by a combination of species with a high cover and differential species that indicate certain conditions. An example decision tree for the Eifel National Park with the dominant grasses *Arrenatherum elatius*, *Cynosurus cristatus*, *Dactylis glomerata*, *Holcus lanatus*, *Festuca rubra agg.* and *Lolium perenne,* and the climbing plants as differential species is shown in Figure 3.

Figure 3. An example decision tree for deriving vegetation types from species composition, the name of species is referring to their simulated cover, first published by Siehoff et al. (2011).

#### Site parameterization – site EIVs

In our approach, species interaction with the environment relies on EIVs (see Section 4.1). This means that a species depends on how well its species EIVs match the corresponding environmental factors, the *site EIVs*. For this interaction to work, it is necessary to provide corresponding scales for site EIVs. Unlike species EIVs (see 5.2.2.3), site EIVs are not generally available. In the past, there have been some attempts to generate site EIVs for different plant communities, e.g. Böcker et al. (1983). However, there are not many sources available for easy reference. Therefore, we provide general guidance on how to obtain site EIVs.

*Briemle site EIVs.* The Briemle species EIVs describe the tolerance of species to three types of land use (utilization): cutting, grazing, and trampling (Briemle et al., 2002). They have been developed mainly for grasslands. The scale ranges from less tolerant (1) to very tolerant (9). For the corresponding site EIVs, Siehoff et al. (2011) developed a relative intensity scale and defined different land use scenarios (Table 6).

Table 6. Land use scenarios with their corresponding utilization intensity were first published by Siehoff et al. (2011).

| Description of the land use form | Utilization intensity  (cutting – grazing – trampling) |
| --- | --- |
| Hay-meadow | 75 – 1 – 1 |
| Mown pasture (very extensive) | 75 – 20 – 10 |
| Extensive pasture | 0 – 35 – 20 |
| Intensive pasture | 0 – 50 – 50 |
| Sheep pen | 0 – 85 – 85 or 0 – 80 –80 |
| Fallow | 0 – 1 – 1 |

*Ellenberg site EIVs.* The original Ellenberg species EIVs describe the optimum of species in their realized niche (Dierschke, 1994). Therefore, we use a different approach than for the Briemle site EIVs and introduce Ellenberg site EIVs as 9-point scales analogous to the original species scales. There are two ways to determine the values at a given site: 1) calculation of average species EIVs based on all mapped species of a vegetation type, and 2) theoretical estimation from other sources such as soil maps with parameters such as elevation, slope, and soil parameters. Both approaches have their advantages and disadvantages (Table 7). The calculation of site EIV from average species EIVs is a common practice in the analysis of vegetation data and is well described (Diekmann, 2003; Dierschke, 1994). Site EIVs derived from a detailed vegetation mapping can indicate very small-scale changes in local conditions. Theoretical estimation of site EIVs requires expert knowledge of phytogeography and is more general because national soil maps are often provided at larger scales and fine-scale maps are not available everywhere. Nevertheless, these soil maps can provide valuable information where meaningful vegetation mapping is not available, such as after a recent disturbance.

Table 7: Advantages and disadvantages of the possible methods for the derivation of Ellenberg site EIVs.

|  | Calculation of average EIVs  based on vegetation mapping and species lists | Theoretical estimation of site EIVs,  based on soil maps |
| --- | --- | --- |
| Advantage | - Fine-scale - Easy to obtain - The principle is well described in the literature - Applicable without further expertise | - Often widely available - Future scenarios use the same logic as current scenarios to derive values |
| Disadvantage | - Not available for future scenarios (only over time for space) - Not available after recent disturbances when the vegetation is destroyed/absent, such as fires, floods, or raw soil conditions - The same sources are used for species EIV selection | - National or federal state-specific standards for soil maps and the available parameters - Requires expert knowledge of phytogeography to determine how soil and site characteristics affect the vegetation |

*Site EIVs based on vegetation mapping.* If detailed vegetation data are available, average site EIVs can be calculated based on the species lists for each vegetation type. This is based on the assumption that the presence of all species in an area reflects the environmental conditions more reliably than a single species. (Diekmann, 2003). Areas are therefore defined and distinguished by the similarity of their species composition. Since the species composition also determines the vegetation type, this is similar to the concept of vegetation types (see 4.1). Therefore, the calculation must be performed separately for each vegetation type with its characteristic species community.

There are several ways to calculate these site EIVs from vegetation data. Either the median or a weighted mean of an EIV can be calculated over all species present. For the weighted mean, there is also a choice between qualitative and quantitative weighting (using presence/absence or abundance data). For a comprehensive analysis and discussion of considerations and pitfalls when calculating weighted average or median EIVs, see Diekmann et al. (2003). Technically, there is no difference in the model depending on which calculation is chosen. It is therefore important that the chosen approach is appropriate for the quality of the available data.

*Site EIVs based on soil map information.* The availability of digital soil maps and the information they provide varies across Germany (Behrens & Scholten, 2006). Here, we provide a compilation of parameters that can be used to estimate site EIVs (Table 8) based on information from German soil maps from North Rhine-Westphalia (NRW) (Schrey, 2014). We have focused on estimating EIVs for open and semi-open landscapes up to high montane altitudes. As a result, not all of the environmental factors, in particular the L- and T-values, are fully available here. Another problem was that the soil maps provide some parameters aggregated in classes. Therefore, it is not always possible to refer to each value on the 9-point scale, and some values are given as proxies for a group.

*Site EIV for light (L-Value).* Ellenberg’s light availability scale for plants ranges from deep shade (1) to partial shade (5) to full light (9) (Ellenberg et al., 1992). To determine the site value from the soil map information, the size of the site, its aspect, and slope can be used (L-Value, Table 8). For an open or semi-open landscape, the L-Values should be 6 or higher. This is because the shaded condition is only temporally. On small sites, the surrounding area may affect the light availability, e.g. due to tree shading. To account for these peripheral effects of the surroundings, we distinguish between small sites (< 5000 m^2^) and large sites (≥ 5000 m^2^) for the L-Value estimation. Small sites generally have a lower value than larger sites with the same parameters. In addition to the site size, aspect, and slope are used to estimate the L-Value. A southern aspect with a moderate slope gradient is beneficial for a high L-Value, while a northern aspect with a steep slope results in a lower L-Value.

For completeness, it should be noted that for a site to be classified as half-shaded or less (1-5), there must be sufficient cover. This may be due to other vegetation, such as a forest, or to special features, such as at the base of a north-facing cliff. A meaningful classification then requires further information to determine the L-Values, such as stem density and forest type.

*Site EIV for temperature (T-Value).* Ellenberg’s temperature scale for plants ranges from plants as cold indicators (1) to extreme heat indicators (9). In Ellenberg et al. (1992), the authors made a first attempt to assign elevation classes and mean annual temperature to the T-Values. Consistent with this, we use the elevation from the soil maps as a parameter for estimating the T-value. Values 8-9 should only be used for warm, lowland sites with and require further information. Similar to the L-Values aspect and hillslope could be used here. To use T-Values below 4, a further adaptation to alpine and subalpine conditions is required.

*Site EIV for soil moisture (M-Value).* Ellenberg’s soil moisture scale for plants ranges from plants as very dry indicators (1) to wet indicators (9) to underwater plants (12). Since the LandS model is designed for terrestrial habitats, the original scale was truncated at 9, to exclude the values dedicated to aquatic plants. German soil maps from North Rhine-Westphalia provide a parameter called “ecological moisture class” (Schrey, 2014). It uses the soil properties usable field capacity in the effective root zone, waterlogging level, and groundwater level to assess the soil moisture. We use this parameter to estimate the M-Value. Other valuable sources could be the slightly different ecological moisture assessments according to Bechler & Toth (2010) or Hauffe et al. (1998).

*Site EIV for soil reaction (R-Value).* Ellenberg’s soil reaction scale for plants ranges from plants as strong acid indicators (1) to alkaline and calcareous soil indicators (9). The soil reaction EIV can be measured by the pH, for example, and is one of the better measurable Ellenberg EIVs (Dierschke, 1994). We used Cation Exchange Capacity (CEC) classes from soil maps to estimate the R-Value. Low CEC values result in low R-Values and vice versa. With more detailed parameter classes, some of the combined categories could be further developed.

*Site EIV for soil nutrient/nitrogen (N-Value).* Ellenberg’s soil nutrient scale for plants ranges from indicators of nutrient-poor sites (1) to indicators of excessively nutrient-rich sites (9). We use soil type, texture, and topsoil thickness from soil maps to estimate how well the soil provides nutrients to plants and derive the N-Value from this estimate. Some soils, such as podzols, regosols, plaggens, syrosems, or rankers, are naturally low in nutrients. For other soil types, texture plays an important role, as coarser textures such as gravel and sand have lower nutrient retention (Gaines & Gaines, 1994; Hassink, 1994). Finally, for very fine-grained soils (clays, class 3-1), the thickness of the soil layer can be used to further segment the N-value.

*Adjusting and adapting site EIVs.* Estimating site EIVs from soil map information may not accurately capture all environmental conditions. Depending on local conditions or the scenario being considered, it may be necessary to modify the values that were estimated above. For example, when simulating used grassland, the effect of fertilization or airborne nitrogen deposition must be considered and the N-value may need to be increased. Small-scale landscape patterns may also require adjustments. For example, it may be necessary to increase the F-Value at the foot of a slope or in depressions; similarly drainage could be accounted for by decreasing the F-Value. This can be done, for example, by using observations or information from a DTM so that the final EIV map reflects the environmental conditions as accurately as possible. In addition, different climate scenarios can be simulated by adjusting the T-Value.

Table 8. Overview of how soil map information is used to derive site EIVs. The parameters provided refer to the information available in the German soil map of NRW, BK50 (GD NRW, 2023; Schrey, 2014).

| **L-Value,** | | | |  | | |  | |  | |  |  | |  |  |  |
| --- | --- | --- | --- | --- | --- | --- | --- | --- | --- | --- | --- | --- | --- | --- | --- | --- |
| **I) Small sites; open area < 5000 m^2^** | |  | | **1 – 5** | | | | |  | | **6** | **6.5** | | **7** | **7.5** | **8 - 9** |
| Aspect | Used for a covered area of any size, e.g. woodlands. Requires further information. | | | | | | | | | | WSW to ESE | Any \| not specified | | WSW to ESE | SE to SW | not defined |
| Hillslope |  |  |  |  |  |  |  |  |  |  | >20° | < 5° \|  not specified | | 5° - 20° | >5° | not defined |
| **II) Large sites; open area ≥ 5000 m^2^** | | **1 – 6** | | | | | | | | |  | **6.5** | | **7.5** | **8** | **9** |
| Aspect | Used for a covered area of any size, e.g. woodlands. Requires further information. | | | | | | | | | | | WSW to ESE | | WSW to ESE | Any | SE to SW |
| Hillslope |  |  |  |  |  |  |  |  |  |  |  | > 20° | | 5° - 20° | < 5°\|not specified | > 5° |
| **T-Value** | **1-3** | | | | | **4** | | **4.5** | | | **5** | **6** | | **7** | **8-9** | |
| Altitude [m] | > 1500 | | | | | 1500-800 | | 800-650 | | | 650-450 | 450-300 | | < 300 | exceptional warm sites, consider aspect | |
| **M-Value** | **1-2 (1.5)** | | | | | **3** | | **4-6 (5)** | | | | | | **7** | **8-9 (8.5)** | |
| Ecological moisture classes | very dry | | | | | dry | | moderately dry - slightly moist | | | | | | moist | wet | |
| **R-Value** | **1-2 (2)** | | **3** | | | **4** | | | | | **5-6 (5.5)** | | | **7-8 (7.5)** | | **9** |
| Classes for cation exchange capacity (CEC) [mol^+^/m^2^] | < 40 | | 40-80 | | | 80-160 | | | | | 160-320 | | | 320-640 | | > 640 |
| **N-Value** | **1-3 (2)** | | **3** | | **3-4 (3.5)** | | | | | **4-6 (5)** | | | **6-7 (6.5)** | | **8-9 (8.5)** | |
| Soil type | P, Q, E, O, N | | R, Z | | B, L, K, S,  SG, G, GN | | | | | B, L, K, S,  SG, G, GN | | | B, L, K, S,  SG, G, GN | | B, L, K, S,  SG, G, GN | |
| Soil textural classes  (of the top layer) | any | | any | | 4, 5, 6, 7, 8, 9 | | | | | 3 | | | 2, 3 | | 1, 2, 3 | |
| Thickness of the top soil layer | any | | any | | any | | | | | < 60 cm | | | 60-200 cm | | > 200 cm | |

Soil type abbreviations: P = podzol; Q = regosol; E = plaggen; O = syrosem; N = ranker; R = rendzina; Z = pararendzina; B = brown earth; L = para-brown earth; K = colluvisol; S = pseudogley; SG = stagnogley; G = gley; GN = wet gley. Soil texture classes: 1 = loamy clay; 2 = clayey loam; 3 = clayey silt; 4 = sandy loam; 5 = very loamy sand; 6 = sandy silt; 7 = loamy sand; 8 = sandy; 9 = low on fine-grained soil.

#### Species parameterization – grouping and species-specific EIVs

Based on the previous analysis of vegetation types, a set of representative species and ecological plant groups can be defined to represent the habitat-specific species composition. First, the species EIVs for site condition and land use are calculated based on literature values, and then the maximum growth rate g_max_ and the self-regulation factor (F_S_) are then calibrated for all species. The calibration follows vegetation patterns that have been identified as being in the equilibrium state (see previous section).

*Set of representative species.* Since modeling of all existing herbaceous species could result in over-parameterization and could make model calibration impossible, a set of representative species can be selected. First, it is important to select the most abundant species that dominate the competition in the study area, as single species. These are often grass species among a few other species, depending on the landscape. It is also useful to select a few characteristic species that are not dominant but are characteristic of certain equilibrium states. Beyond these single species, the remaining species can be grouped into plant groups of species with similar ecological behavior. These groups usually need to be adapted to the set of environmental factors that drive the specific landscape. For example, when studying managed grasslands, traits that integrate a response to mechanical disturbance by cutting or grazing (Briemle et al., 2002) may be useful. However, in wetlands, for example, other factors such as different moisture or nutrient groups may be more appropriate.

*Species EIVs.* Values for the different species can be found in the literature. Ellenberg’s EIVs for light, temperature, moisture, soil reaction (pH), and nutrients are published for Germany in Ellenberg et al. (1992, 2001) and Ellenberg & Leuschner (2010). Ellenberg-type EIVs are also available for other European countries and on a European scale (Tichý et al., 2023). A European map with recommendations for areas where these Ellenberg-type values can be applied is also provided by Tichý et al. (2023). Briemle’s EIVs for cutting, grazing, and trampling are published in Dierschke & Briemle (2002). EIVs of species groups can be represented either by a group indicator species and its literature values, or by an average of all species in the group and their literature values. If an average of all species in a group is calculated, it may be necessary to introduce a threshold for the degree of occurrence to exclude randomly occurring species. Calibration results can be enhanced during the pre-configuration process by adjusting the EIVs of some species to the simulated geographic area according to the obtained data at the study site and expert knowledge as suggested by Ellenberg et al. (1992), Dierschke (1994), and Briemle et al. (2002), within a maximum change of +/- 0.2.

*Calibrating the g_max_ and F_S_ parameters.* The iterative calibration process involves adjusting the parameters g_max_ and F_S_ for each plant species to visually match the simulated cover with the predefined cover of different calibration scenarios. These calibration scenarios are usually vegetation types in the equilibrium state under the influence of different environmental factors, and sometimes short-term developments from one vegetation type to another after a change in environmental conditions. The procedure starts by simulating a single scenario and adjusting the parameters until the simulated vegetation composition matches the observed reference values (Figure 4).


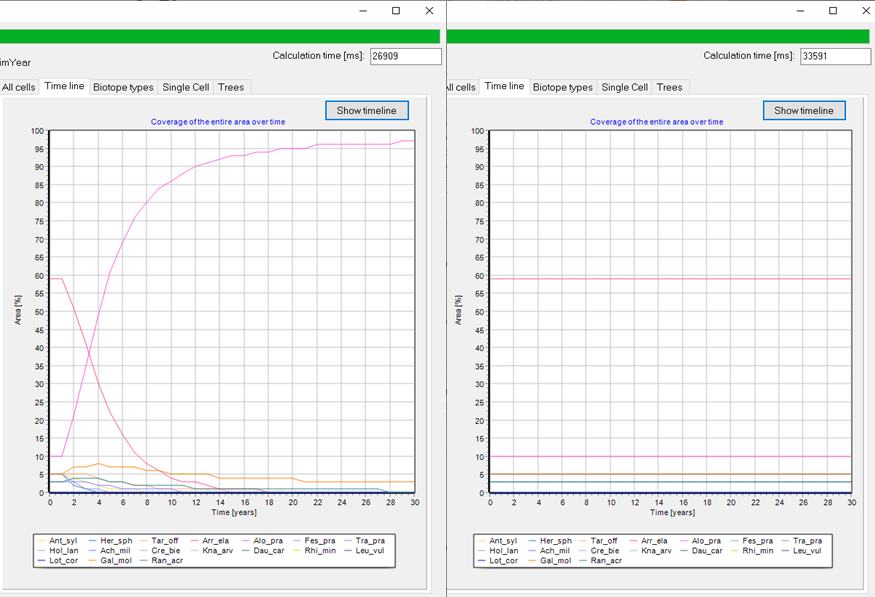


Figure 4. Screenshots showing parts of the output GUI of two different simulation runs: at the start of the calibration process (left) and after calibration (right) with the dynamic equilibrium of the examined community.

Subsequent scenarios are calibrated in the same way, but with a backward check to ensure that previously calibrated scenarios are still accurate. The parameters need to be fine-tuned, especially for species that are present in multiple calibration scenarios, to prevent the species cover from evolving too far from reality. Before calibration, it is helpful to categorize species based on the patterns of dominance and abundance observed in the scenarios (Table 9). Species with high cover tend to be dominant and thus have high F_S_ values. Species with low abundance often have a low g_max_ and are therefore less competitive in the community in a variety of scenarios. Once a few scenarios have been calibrated, this should become easier. If the calibration or backward checks are difficult, it may be necessary to change the weighting of the environmental factors (see below) to better represent the dominant environmental gradients of the simulated sites. The calibrated g_max_ and F_S_ values of all species are then used for all simulations of the given landscape.

Table 9. Categorization of observable patterns to derive exemplary start values for calibration.

| Observable pattern | Calibration values |
| --- | --- |
| Dominant species | F_s_= 1000 |
| Less dominant species | F_s_ = 500 |
| Least dominant species | F_s_ = 100 |
| Species with a high abundance | g_max_ = 5.0 |
| Species with a medium abundance | g_max_ = 4.5 |
| Species with a low abundance | g_max_ = 3.5 |

*Weighting of environmental factors*. The weights of the environmental factors are important to regulate the impact that a factor has on the growth of species (see 7.1.3.2). While it is possible and sometimes necessary to tweak the weighting values during the pre-configuration process, the weights should depict the important gradients at the studied site. For example, modeling grasslands along a moisture gradient may benefit from a higher weight for moisture, or modeling a forested landscape may make light availability more important and thus more heavily weighted, while other factors such as soil pH, if constant in these landscapes, are less heavily weighted because they do not play a strong role in differentiating the vegetation. Observations and data analyses of the observed species with their species EIVs at different sampling sites within the landscape can help evaluate the importance of different factors.

# Input data

The model does currently not include input data that is loaded during the simulation run.

# Submodels

As the model contains mandatory and optional submodels for vegetation and disturbance elements, we use a nested ODD approach here to describe each submodel in detail if used.

## Herbaceous vegetation

The model for the forb and grass layer was first developed by Siehoff et al. (2011) and used as a submodel by Hudjetz et al. (2014). Here we extend the existing model with a second set of growth dependencies.

### Purpose

The forb and grass layer submodel is the foundation of the landscape and thus the underlying component for all subsequent succession. The model simulates vegetation growth based on environmental conditions and disturbances.

### Process overview and scheduling

The herbaceous submodel is embedded in each cell of the landscape grid to calculate species’ cover. All cells are recalculated each time step from the upper left corner to the lower right one. At each time step within all cells, each plant behaves in the following order (for an overview see Figure 2, green boxes represent processes of the herbaceous vegetation):

First, a certain part of the cover of each plant dies creating available space within the cell. Mortality is implemented to allow the processes of competition to take place even when the whole space is used up. Without mortality, the plants could not grow (and thus compete) as soon, as the whole area is covered. The plants are not able to push each other out, but gain competitive power only by their realized growth under competition with the other plants into available (uncovered) space.

The growth of the species is then calculated as a function of its cover, its sensitivity to the given environmental conditions and land use (using EIVs), its maximal growth rate, and its factor for self-regulation. A part of the species’ growth is transferred to the adjacent cells, simulating vegetative spread; seed dispersal over larger distances is not taken into account. This ingrowth into neighboring cells results either in additional growth of an already existing species or in a new species immigrating into the cell. If the sum of potential growth and ingrowth from neighboring cells of all species exceeds the available space within the cell, the available area is divided according to the species’ potential growth, and a new realized growth is calculated. The competition between the different species takes place only in this step when the potential growth is recalculated to realized growth. The competitive strength of each plant is derived from the dynamic growth model by the relative weighting of the potential growth of each species.

At the end of each time step, the cover of all species is updated. From the cover of the dominant plants, the vegetation type of the cell is derived, which can be plotted into raster-maps.

### Herbaceous vegetation submodel

The simulation of herbaceous species is divided into four submodels: a constant mortality model, a dynamic growth model that calculates the potential growth of the species based on its growth rate, and the competition model. These submodels are described in the following sections.

Table 10. Equations used in the herbaceous vegetation submodel.

| Eq.# | Description | Function | Source |
| --- | --- | --- | --- |
| 1 | Mortality | M = cover × 200% a^-1^ | (Siehoff et al., 2011) |
| 2 | Growth rate | $g=g_{max}\times\frac{F_{s}-\left( \frac{c}{AS} \right)\times100}{F_{s}}\times f_{total}$ | (Siehoff et al., 2011), this paper |
| 3 | Control function for land use X and species j | $f\left( x \right)=\left( 1-\frac{EIV_{x_{j}}}{9} \right)\times\frac{100-{SV}_{x}}{100}+\frac{EIV_{x_{j}}}{9}$ | (Siehoff et al., 2011) |
| 4 | Control function for site condition X and species j | $f\left( x \right)=\left\{ \begin{aligned} normal EIV,e^{-\mathrm{slope}_{x}\frac{\left( {SV}_{x}- {EIV}_{x_{j}} \right)^{2}}{9}} \\ &indifferent EIV,e^{-\mathrm{slope}_{x}\frac{\left( {SV}_{x} -{EIV}_{x_{j}} \right)^{2}}{9}}-{Opt}_{x} \end{aligned} \right.$ | This paper |
| 5 | Weighting of control functions | $W_{x}=\left( 2-f\left( x \right) \right)^{w_{x}}$ | This paper |
| 6 | Total environmental dependency function | $f_{total}=\frac{\sum\left( W_{x}\times f\left( x \right) \right)}{\sum x - \sum W_{x}}$ | This paper |
| 7 | Potential growth | $\left( \frac{dc}{dt} \right)_{pot}=g\times c$ | (Siehoff et al., 2011) |
| 8 | Realized growth | $\left( \frac{dc}{dt} \right)_{real}=g\times c\times\frac{available space}{\sum\left( \frac{dc}{dt} \right)_{pot}}$ | (Siehoff et al., 2011) |

#### Mortality

A fixed arbitrary mortality rate of 200% per year for all species is assumed to create available space. To obtain the corresponding mortality for each growth cycle, the annual mortality is divided by time steps. At the beginning of each growth cycle, the cover of each entity is reduced accordingly.

#### Dynamic growth model

##### Calculation of growth rate

The growth rate of each plant species is calculated according to its current cover, its maximal growth rate, its factor for self-regulation, and the local environmental dependencies (Eq. 1).

The values for maximum growth rate and factor for self-regulation need to be calibrated on vegetation data from the area under study. More information about data and scenarios used for calibration is given in Section Species parameterization (5.2.2.3).

The environmental dependencies on the EIVs are implemented as two different types of control functions: the site condition control function and the land use control function. These control functions are then weighted to represent the importance of each environmental factor to the simulated landscape and the study site. Finally, an overall environmental growth factor is calculated from the weighted growth functions. How environmental factors influence growth rate has changed from the previous model version with the introduction of the second set of EIVs (see below). For a detailed description of how the species EIVs are obtained see Section 5.2.2.3 (Species parameterization).

*Control function for site conditions*

The control functions for site conditions are calculated as ecological tolerance functions (Eq. 4). The optimum value of 1 is reached when a species’ EIV is equal to the corresponding site EIV. Suboptimal conditions are indicated by values below 1 (see Figure 5 A). In addition, the slope regulates the width of the optimum curve, increasing or decreasing the sharpness of the ecological tolerance to an environmental condition. It therefore determines how strong the growth factor is reduced when species EIV and site EIV are not equal. In case an EIV is indifferent for a certain species, we modified the regular control function with the possibility to reduce the slope and the optimum. The reduced slope mimics the broader tolerance of the species. These modifications, as well as the regular slope, are specific to each EIV, but globally valid for all species.

*Control function for land use*

The control functions for the form of land use are calculated as linear sensitivity functions (Eq. 3). The degree of growth inhibition depends on the intensity of land use. The function *f*(X) results in values between 0.1 and 1, decreasing the growth rate of species that are sensitive to the given land use (Figure 5 B). A value of 1 signifies that the species is not affected by the form of land use.


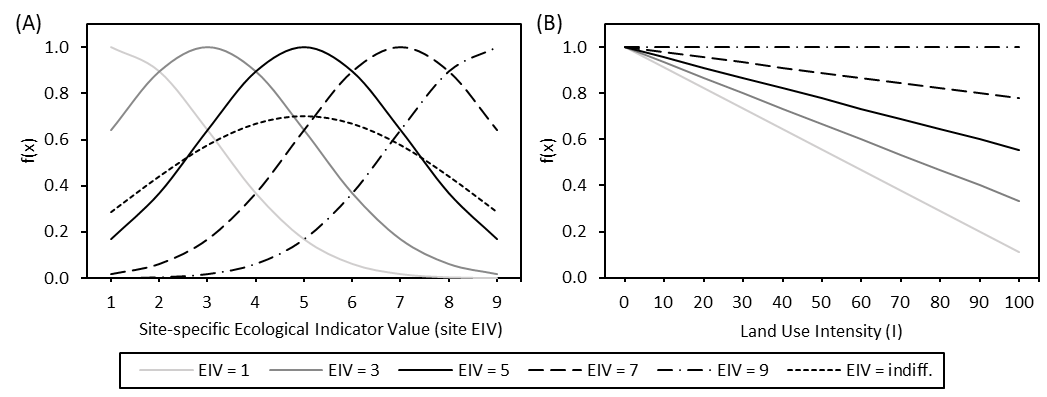


Figure 5. Control functions for site conditions (A) and land use (B) at different species EIVs. (A) Control functions are calculated here for regular species EIVs with a slope of 1, and for indifferent species EIVs with a reduced slope of 0.3 and a reduced optimum of 0.6. (B) There are no indifferent species for the land use.

*Total environmental dependence function and weights*

In an earlier version of the model, we calculated the environmental dependence by simply multiplying all the values of the environmental control functions. Typically, however, environmental dependencies are at suboptimal conditions, i.e., below values of 1. With three control functions, a typical value would be 0.7^3^ ≈ 0.34, but introducing five more factors shifts the value in the example to 0.7^8^, resulting in a total value <0.1. This issue has previously been discussed in a forest gap model (Bugmann, 1994). Therefore, we introduce here an alternative way to calculate the total environmental factor (Eq. 6). This approach uses the weighted means of the control functions (Eq. 5). It also introduces the possibility of weighting individual factors according to their ecological relevance to the current landscape and study site. These weights are global within the modeled environment and therefore the same for all species. They can be adjusted for different landscapes and are assessed during pre-configuration (see Section 5.2.2.3). When weighted equally, environmental factors play similar roles in the growth of species. When individual factors are weighted significantly higher, the growth curve shifts toward adaptation to those factors. This flexibility allows better calibration for a variety of landscapes with different dominating gradients.

##### Calculation of potential growth

The total potential growth of each species is the sum of potential growth within a cell and ingrowth from adjacent cells. Potential growth is calculated according to the species’ growth rate and its cover (Eq. 7). This potential growth refers to the capability of a species to grow into an uncovered area. Since each cell is a homogeneous entity and no information about the distribution of species within one cell is given, potential growth is randomly split into the potential growth within the actual cell and to adjacent cells (section 4.6), where it is added to the total potential growth of the plant species.

#### Competition model - calculation of realized growth

Plant species interact by struggling for the available space. The share of available space that each species gets depends on its potential growth compared to the others. If the sum of the potential growth of all species exceeds the available area, the area is divided among the species according to their potential growth, i.e. the sum of growth within the cells and ingrowth from adjacent cells (Eq. 8).

This function simulates a ‘simultaneous’ growth of all species and their competition for space. The species with the highest potential growth will occupy the largest fraction of the available area, where potential growth is dynamically calculated as a function of cover, maximum growth rate, intraspecific competition, and sensitivity to the given environmental conditions (7.1.3.2).

This competition model acts in a way that the competitive vigor of a species does not only depend on its intrinsic strength, indicated by the parameters g_max_ and F_S_, but is strongly influenced by its tolerance of the prevailing environmental conditions as well as the applied form of land use. Two species with the same values for g_max_ and F_S_, but different EIVs will therefore behave differently according to the environmental factors.

## Woody vegetation

*Woody vegetation.* Optional and not used in the current model setup.

## Disturbance by wildlife

Disturbance by wildlife is used for *woody vegetation.* Therefore, it is not relevant to the current model setup.

# References

Bechler, K., & Toth, O. (Eds.). (2010). *Bewertung von Böden nach ihrer Leistungsfähigkeit: Leitfaden für Planungen und Gestattungsverfahren* (2., völlig überarb. Neuaufl). Landesanstalt für Umwelt, Messungen und Naturschutz Baden-Württemberg.

Behrens, T., & Scholten, T. (2006). Digital soil mapping in Germany—A review. *Journal of Plant Nutrition and Soil Science*, *169*(3), 434–443. https://doi.org/10.1002/jpln.200521962

Bithell, M., & Macmillan, W. D. (2007). Escape from the cell: Spatially explicit modelling with and without grids. *Ecological Modelling*, *200*(1–2), 59–78. https://doi.org/10.1016/j.ecolmodel.2006.07.031

Böcker, R., Kowarik, I., & Bornkamm, R. (1983). Untersuchungen zur Anwendung der Zeigerwerte nach Ellenberg. *Verhandlungen Der Gesellschaft Für Ökologie*, *11*, 35–56.

Braun-Blanquet, J. (1964). *Pflanzensoziologie*. Springer Vienna. https://doi.org/10.1007/978-3-7091-8110-2

Briemle, G., Nitsche, S., & Nitsche, L. (2002). Nutzungswertzahlen für Gefässpflanzen des Grünlandes. *Bundesamt für Naturschutz Bonn*, *38*(2), 203–225.

Bugmann, H. (1994). *On the Ecology of Mountainous Forests in a Changing Climate: A Simulation Study*. ETH Zurich.

Chesson, P. L., & Warner, R. R. (1981). Environmental Variability Promotes Coexistence in Lottery Competitive Systems. *The American Naturalist*, *117*(6), 923–943. https://doi.org/10.1086/283778

Diekmann, M. (2003). Species indicator values as an important tool in applied plant ecology – a review. *Basic and Applied Ecology*, *4*(6), 493–506. https://doi.org/10.1078/1439-1791-00185

Dierschke, H. (1994). *Pflanzensoziologie: Grundlagen und Methoden*. Ulmer.

Dierschke, H., & Briemle, G. (2002). *Kulturgrasland: Wiesen, Weiden und verwandte Staudenfluren; 20 Tabellen*. Ulmer.

Ellenberg, H., & Leuschner, C. (2010). *Vegetation Mitteleuropas mit den Alpen: In ökologischer, dynamischer und historischer Sicht; 203 Tabellen* (Freds Büro; 6., vollst. neu bearb. und stark erw. Aufl). Ulmer.

Ellenberg, H., Weber, H. E., Düll, R., Wirth, V., Werner, W., & Paulißen, D. (2001). *Zeigerwerte von Pflanzen in Mitteleuropa* (Flur; 3. durchgesehene Auflage, Vol. 18). Erich Goltze KG.

Ellenberg, H., Weber, H. E., Düll, R., Wirth, V., Werner, W., & Pauliβen, D. (1992). *Zeigerwerte von Pflanzen in Mitteleuropa* (2. verbesserte und erweiterte Auflage, Vol. 18). Erich Goltze KG.

Gaines, T. P., & Gaines, S. T. (1994). Soil texture effect on nitrate leaching in soil percolates. *Communications in Soil Science and Plant Analysis*, *25*(13–14), 2561–2570. https://doi.org/10.1080/00103629409369207

GD NRW. (2023). *Bodenkarte von Nordrhein-Westfalen 1: 50 000 (BK 50) WMS* [Map]. Geologischer Dienst NRW. https://www.wms.nrw.de/gd/bk050

Grimm, V., Railsback, S. F., Vincenot, C. E., Berger, U., Gallagher, C., DeAngelis, D. L., Edmonds, B., Ge, J., Giske, J., Groeneveld, J., Johnston, A. S. A., Milles, A., Nabe-Nielsen, J., Polhill, J. G., Radchuk, V., Rohwäder, M.-S., Stillman, R. A., Thiele, J. C., & Ayllón, D. (2020). The ODD Protocol for Describing Agent-Based and Other Simulation Models: A Second Update to Improve Clarity, Replication, and Structural Realism. *Journal of Artificial Societies and Social Simulation*, *23*(2). https://doi.org/10.18564/jasss.4259

Gross, L. J., & DeAngelis, D. (2002). Multimodeling: New approaches for linking ecological models. *Predicting Species Occurrences: Issues of Accuracy and Scale*, 467–474.

Hassink, J. (1994). Effects of soil texture and grassland management on soil organic C and N and rates of C and N mineralization. *Soil Biology and Biochemistry*, *26*(9), 1221–1231. https://doi.org/10.1016/0038-0717(94)90147-3

Hauffe, H.-K., Augenstein, I., Vogelgsang, W., & Lehle, M. (1998). Bewertung von Böden als „Standort für die natürliche Vegetation" In: Natursc hutz und Landschaftsplanung 30(7), 214 -219. *Naturschutz Und Landschaftsplanung*, *30*(7), 214–219.

Hudjetz, S., Lennartz, G., Krämer, K., Roß-Nickoll, M., Gergs, A., & Preuss, T. G. (2014). Modeling Wood Encroachment in Abandoned Grasslands in the Eifel National Park – Model Description and Testing. *PLOS ONE*, *9*(12), e113827. https://doi.org/10.1371/journal.pone.0113827

Knapp, R. (1974). Cyclic Successions and Ecosystem Approaches in Vegetation Dynamics. In R. Knapp (Ed.), *Vegetation Dynamics* (pp. 91–100). Springer Netherlands. https://doi.org/10.1007/978-94-010-2344-3_10

Müller, J., & Rosenthal, G. (1998). Brachesukzessionen—Prozesse und Mechanismen. *Berichte des Instituts für Landschafts- und Pflanzenökologie*, *5*, 103–132.

Pickett, S. T. A. (1989). Space-for-Time Substitution as an Alternative to Long-Term Studies. In G. E. Likens (Ed.), *Long-Term Studies in Ecology: Approaches and Alternatives* (pp. 110–135). Springer. https://doi.org/10.1007/978-1-4615-7358-6_5

Prach, K., & Řehounková, K. (2006). Vegetation succession over broad geographical scales: Which factors determine the patterns? *Preslia*, *78*(4), 469–480.

Rademacher, C., Neuert, C., Grundmann, V., Wissel, C., & Grimm, V. (2004). Reconstructing spatiotemporal dynamics of Central European natural beech forests: The rule-based forest model BEFORE. *Forest Ecology and Management*, *194*(1–3), 349–368. https://doi.org/10.1016/j.foreco.2004.02.022

Rammig, A. (2005). *Disturbance in mountain forests: Analysing, modelling and understanding successional processes after blowdown events* (p. 150 p.) [ETH Zurich; Application/pdf]. https://doi.org/10.3929/ETHZ-A-005162359

Schreiber, K.-F. (1997). Characteristics of succession in 20 years’ abandoned grassland studies in Baden-Württemberg. *Forstwissenschaftliches Centralblatt*, *116*(1), 243–258. https://doi.org/10.1007/BF02766901

Schrey, H. P. (2014). *Bodenkarte von Nordrhein-Westfalen 1:50.000: BK 50 ; [Inhalt, Aufbau, Auswertung]*. Geologischer Dienst Nordrhein-Westfalen.

Siehoff, S. (2011). *Succession of semi-natural grasslands: Spatially-explicit, mechanistic simulation considering various forms of land use* [Dissertation]. RWTH Aachen University.

Siehoff, S., Lennartz, G., Heilburg, I. C., Roß-Nickoll, M., Ratte, H. T., & Preuss, T. G. (2011). Process-based modeling of grassland dynamics built on ecological indicator values for land use. *Ecological Modelling*, *222*(23), 3854–3868. https://doi.org/10.1016/j.ecolmodel.2011.10.003

Silvertown, J. (2004). Plant coexistence and the niche. *Trends in Ecology & Evolution*, *19*(11), 605–611. https://doi.org/10.1016/j.tree.2004.09.003

Smit, R., & Olff, H. (1998). Woody species colonisation in relation to habitat productivity. *Plant Ecology*, *139*(2), 203–209.

Tichý, L., Axmanová, I., Dengler, J., Guarino, R., Jansen, F., Midolo, G., Nobis, M. P., Van Meerbeek, K., Aćić, S., Attorre, F., Bergmeier, E., Biurrun, I., Bonari, G., Bruelheide, H., Campos, J. A., Čarni, A., Chiarucci, A., Ćuk, M., Ćušterevska, R., … Chytrý, M. (2023). Ellenberg-type indicator values for European vascular plant species. *Journal of Vegetation Science*, *34*(1), e13168. https://doi.org/10.1111/jvs.13168

Vincenot, C. E., Giannino, F., Rietkerk, M., Moriya, K., & Mazzoleni, S. (2011). Theoretical considerations on the combined use of System Dynamics and individual-based modeling in ecology. *Ecological Modelling*, *222*(1), 210–218. https://doi.org/10.1016/j.ecolmodel.2010.09.029

Zolotova, E., Ivanova, N., & Ivanova, S. (2023). Global Overview of Modern Research Based on Ellenberg Indicator Values. *Diversity*, *15*(1), Article 1. https://doi.org/10.3390/d15010014
